# Sex-differences in the intergenerational transmission of mental disorders among schizophrenia probands: familial risk and protective factors in a population-based study

**DOI:** 10.1016/j.lanwpc.2025.101750

**Published:** 2025-11-20

**Authors:** Zhi Sheng, Tianhang Zhou, Chunyu Du, Tingfang Wu, Liping Wen, Xianmei Yang, Wencai Chen, Xuehong Ma, Hua Deng, Ling Ge, Changchun Zhang, Xu Hong, Rui He, Xiangdong Du, Lingyan Zhu, Hu Xiang, Sijing Chen, Jiachen Liu, Yongzhuo Ding, Guangming Liang, Yang Pan, Shujiao Ji, Zhengjiao Chang, Nian Yuan, Weiran Chen, Xiaozhen Lv, Hong Ma, Lili Guan, Xin Yu

**Affiliations:** a Peking University Sixth Hospital, Peking University Institute of Mental Health, NHC Key Laboratory of Mental Health (Peking University), National Clinical Research Centre for Mental Disorders (Peking University Sixth Hospital), Beijing, China; b Mental Health Guidance Centre, Beijing Haidian Psychological Rehabilitation Hospital, Beijing, China; c Department of Psychiatry, Beijing Anding Hospital, Capital Medical University, Beijing, China; d Department of Preventive Medicine, Zigong Mental Health Centre, Zigong, China; e Department of Psychiatry, The Third Hospital of Mianyang, Mianyang, China; f Department of Psychiatry, Affiliated Wuhan Mental Health Centre, Tongji Medical College of Huazhong University of Science and Technology, Wuhan, China; g Department of Psychiatry, Baoji Mental Health Center, Shaanxi, China; h Department of Psychiatry, Changchun Sixth Hospital, Changchun, China; i Free Drug Treatment Department, Shenyang Mental Health Centre, Shenyang, China; j Department of Psychiatry, Fangshan Psychiatry Hospital, Beijing, China; k Department of Mental Health, Xiamen Xianyue Hospitial, Xiamen, China; l Department of Psychiatry, Suzhou Guangji Hospital, Suzhou, China

**Keywords:** Mental health transmission, Schizophrenia, Developmental psychopathology, Parental-sex disparities, China

## Abstract

**Background:**

Parental schizophrenia confers intergenerational mental health risks. The sex-specific transmission patterns remain poorly quantified. This study examined parent-reported transmission rate and familial risk factors among Chinese offspring of schizophrenia probands.

**Methods:**

In this cross-sectional study, we enrolled 27,315 schizophrenia probands and 35,772 offspring. Psychiatric disorder diagnoses of offspring were parent-reported and subsequently verified via the medical management system. Age- and sex-standardized rate referenced China's 2020 census. Robust Poisson regression generated adjusted rate ratios (aRR). Multivariable logistic models were used to identify risk factors for young offspring. This study was registered with ClinicalTrials (NCT07005245).

**Findings:**

The parent-reported transmission rate in offspring was 2.68% (95% CI, 2.52%–2.85%), dominated by schizophrenia spectrum disorders (1.42%). Standardized rates reached 2.53% (95% CI, 2.01%–3.05%) after demographic standardization. Offspring risk increased by 86.0% when conception occurred post-parental illness onset. Firstborn status (aRR = 1.67), low-income household (aRR = 1.43), and being a male child (aRR = 1.14) were significantly associated with elevated risk. Sex-specific parental age effects were also observed. In the subgroup analysis of underage (<17 years) offspring, maternal transmission was associated with post-parental-onset birth (OR = 2.48, *p* < 0.001), lower household income (OR = 2.32, *p* < 0.001), and prenatal antipsychotic exposure (OR = 1.69, *p* < 0.001). Paternal transmission was related to father-only caregiving (OR = 2.15, *p* < 0.001), lower household income (OR = 1.97, *p* < 0.001), and male offspring (OR = 1.79, *p* = 0.001). Dual-parent care demonstrated a robust protective effect across both maternal and paternal schizophrenia groups (OR = 0.75, 95% CI, 0.51–1.10).

**Interpretation:**

Our findings highlighted critical interactions between familial aggregation, sex-differentiated perinatal exposures, and caregiving environments. Policy priorities should integrate sex-stratified genetic counselling, prenatal medication monitoring, and family support programs targeting caregiving inequalities.

**Funding:**

This study was supported by the 10.13039/501100001809National Natural Science Foundation of China (32070589).

**Research in context**

**Evidence before this study**

We systematically reviewed PubMed (inception to May 2025) using the terms “(intergenerational transmission) AND (parental schizophrenia) AND (mental disorders)”, supplemented by manual screening of references. Prior evidence confirmed that parental schizophrenia elevates offspring psychiatric risks, but with unresolved gaps. Existing studies rarely disaggregated maternal vs. paternal transmission or offspring sex differences, despite biological and social pathways likely differing by sex. While caregiving environment was theorized to modulate risk, no large-scale studies had quantified the protective potential of dual-parent care—especially in non-Western contexts like China, where multigenerational caregiving norms prevailed.

**Added value of this study**

This study of 27,315 schizophrenia probands and 35,772 offspring provided the first nationally representative, clinically verified estimates of mental health transmission in China. A standardized transmission rate of 2.68% was dominated by schizophrenia spectrum disorders (1.42%), with underreported mood disorders suggesting detection gaps for milder conditions. Maternal transmission strongly tied to post-diagnosis birth, poverty, and prenatal antipsychotic exposure, while paternal transmission was driven by father-only caregiving and male offspring. Additionally, dual-parent care was a robust protective effect across both maternal and paternal schizophrenia groups, highlighting caregiving environment as a modifiable target for intervention.

**Implications of all the available evidence**

Our findings demand a paradigm shift from generic risk counseling to precision prevention. Clinical practice should integrate sex-specific genetic counselling (e.g., tailored discussions of paternal vs. maternal risk pathways) and prenatal monitoring of antipsychotic use, particularly in low-income households. Poverty-alleviation programs should integrate mental health components, given its multiplicative risk effect. In China and similar settings, interventions should leverage existing family structures (e.g., extending multigenerational care support) while addressing gender-specific vulnerabilities.

## Introduction

Schizophrenia imposes a disproportionate global disease burden, affecting 0.3%–0.7% of the population worldwide with particularly profound consequences in low- and middle-income countries (LMICs), where over 80% of affected individuals lack access to adequate treatment.^1^^,^^2^ Accumulating evidence confirms elevated morbidity among offspring of individuals living with schizophrenia compared to the general population.^3^^,^^4^ Family dysfunction, socioeconomic deprivation, and stigma contribute to a rearing environment that marginalizes children's needs,^5^ with stigma-induced silence exacerbating unnoticed mental problems.^6^ However, due to inadequacies in mental health service framework and slow translation of research findings into clinical care, these vulnerable offspring often fail to be identified as early as feasible, with consequences of delayed intervention and unfavorable prognosis.^4^ In China, where there are over 6 million individuals living with schizophrenia,^7^ the intergenerational consequences remain underexplored.

The intergenerational transmission of schizophrenia involves multidimensional mechanisms, which are inadequately addressed in clinical practice.^3^^,^^4^ While genetic factors drive neurodevelopmental anomalies,^8^ environmental modifiers—such as immigration status, obstetric or perinatal complications, and childhood adversities—significantly influence psychosis onset in familial high-risk populations.^9^ Indeed, parental severe mental illness (SMI) itself represents a profound childhood adversity, intersecting with parental neglect and household dysfunction.^10^ Additionally, the gene-environment interplay may operate via epigenetic mechanisms.^11^ Prenatal exposures modulate neurodevelopmental pathways,^12^ while postnatal stressors disrupt attachment and cognitive development.^13^

Examining intergenerational transmission pathways provides critical insights into early psychopathology manifestation. Moreover, a deeper and clinically focused understanding of the intergenerational factors that contribute to predisposition to SMI is a key step in advancing early preventive efforts.^14^ Current epidemiological estimates predominantly derive from Western populations,^15^ with critical gaps in cross-population comparisons and insufficient attention to Asian-specific modifiers. Most studies focus on schizophrenia spectrum disorders despite meta-analytic evidence of elevated transdiagnostic risks in offspring.^16^ Furthermore, parental-sex differentiated transmission remains unexamined, despite paternal care deficits and maternal prenatal pharmacotherapy risks observed preliminarily.^17^

Therefore, we carried out a national cross-sectional study to investigate childbearing behaviors and intergenerational transmission risks of SMI in China. Using parent-reported data, this study presented a subgroup analysis to quantify age-sex-standardized rates of mental disorders among offspring of Chinese parents with schizophrenia. We further explored risk and protective factors across developmental-stages and parental-sex differentiated risk architectures, thereby identifying targets for breaking the cycle of intergenerational mental illness in China.

## Methods

### Study design and participants

**Fig. 1:**
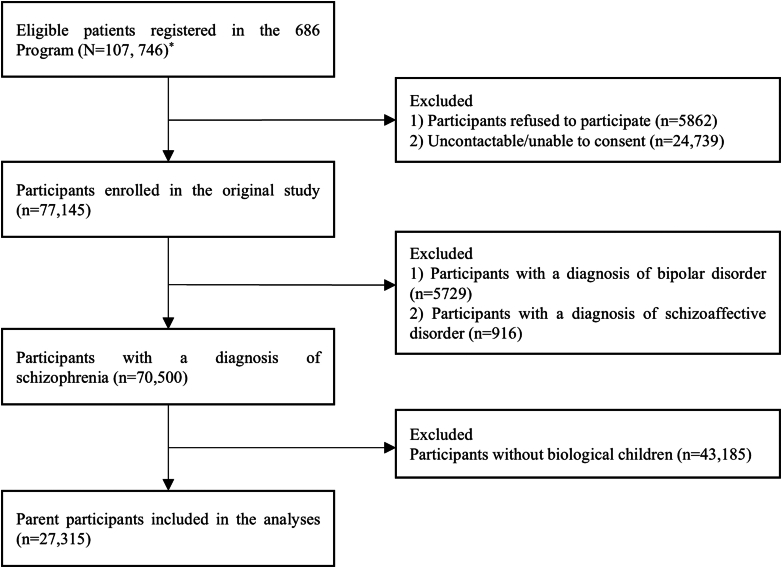
**Participant flow.** Note: ∗ A total of 107,746 participants met the inclusion criteria of the original study were identified from the 686 Program. They were aged 18–59 years, clinically diagnosed with schizophrenia, bipolar disorder, or schizoaffective disorder, and had engaged in continuous community services for more than 6 months in the 686 Program.

**Fig. 2:**
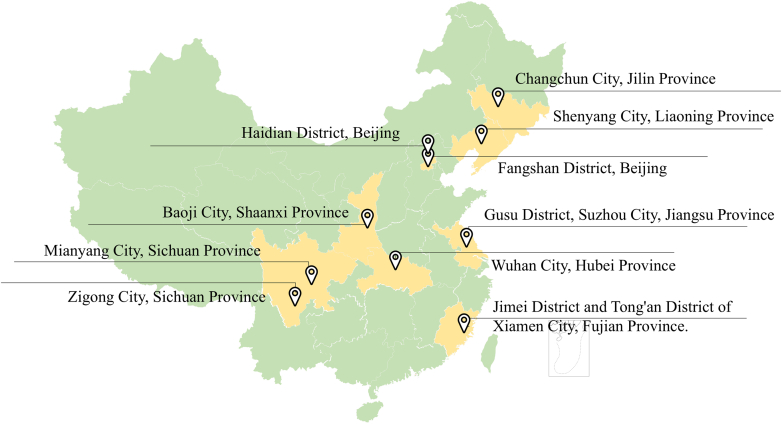
**Study sites**.

The participant flow is depicted in Fig. 1. This study was conducted between June 2021 and October 2022 across 10 cities/districts nationwide (Fig. 2). The study sites were selected through a dual-strategy approach combining geographic-economic stratification and operational feasibility. All prefecture-level administrative units were first stratified by region (eastern/central/western) and economic status (GDP tertiles). Within this framework, priority was given to areas where our research team had established collaboration networks through prior implementation of the National Continuing Management and Intervention Program for Psychosis (also known as the 686 Program). Briefly, the 686 Program which is a core component of China's national mental health management system is specifically designed to deliver standardized, regular community-based mental health services for patients with SMI, including schizophrenia, bipolar disorder, schizoaffective disorder, delusional disorder, and epilepsy-related mental disorders.^18^ Patients undergo a definitive diagnosis conducted jointly by a licensed psychiatrist and an attending psychiatrist, either in psychiatric outpatient or inpatient settings, prior to registration in the system.

This study focused on participants diagnosed with schizophrenia who had biological offspring. Eligibility required definitive schizophrenia diagnosis; at least one biological child documented in household registration systems; and continuous engagement in the 686 Program community-based services for over 6 months preceding the study. Participants with schizophrenia unable to independently consent or participants without children were excluded. The final sample included 27,315 participants with 35,772 offspring data.

### Procedures

Eligible participants were identified from the 686 Program registry, with a psychiatrist-confirmed diagnosis of schizophrenia based on ICD-10 criteria. Community mental health workers (CMHWs) within the designated catchment area conducted face-to-face interviews at community health centers to collect data via a secure Electronic Data Capture (EDC) platform. The platform featured encrypted data transmission, multi-level user authentication, and automated logic checks to ensure data accuracy. For participants with limited digital access, CMHWs performed home visits to collect data using mobile-assisted entry.

Offspring psychiatric diagnoses were reported by their parents during in-person inquiries about the offspring's past/current mental health conditions. These parent-reported diagnoses then underwent a tiered verification protocol tailored to disorder type. Severe mental disorders (e.g., schizophrenia, bipolar disorder) was confirmed by cross-referencing parent reports with the 686 Program registry. Non-psychotic conditions (e.g., depression, autism spectrum disorder) required validation through medical records from clinical encounters. Cases without medical records were retained in the dataset but categorized as unclear diagnoses, with corresponding rate estimates reported separately.

A comprehensive quality assurance framework was implemented. Before data collection, all researchers and CMHWs completed an 8-h training program at PUIMH, which covered EDC system operation, ethical engagement with vulnerable populations, and diagnostic verification protocols. Throughout the study, researchers from PUIMH maintained ongoing communication with local teams to address operational issues. Quality control measures included regular randomized audits, where supervisors independently rechecked 10% of data entries. Data verification involved: (a) extraction of registry-based diagnoses and demographics, and (b) telephone verification of childbearing history and children's mental health with participating families. All telephone interviews were conducted by trained researchers using PUIMH-approved protocols.

#### Measurements

The primary outcome was the rate of parent-reported, protocol-verified mental disorder diagnoses among offspring. Key exposure variables and covariates included: 1) Parental sociodemographic: marital status and per capita household income per capita; parental age at schizophrenia onset and its temporal relationship to childbirth (pre/post-delivery), ascertained via parental self-report cross-checked against medical records from the 686 Program; 2) Offspring environment: primary caregiver status and perinatal health indicators (medication use during pregnancy), obtained through parental recall. All variables were collected using standardized questions embedded in the EDC system, with skip-logic ensuring internal consistency. CMHWs received protocol-specific guidance to clarify ambiguities during data collection, and PUIMH researchers conducted weekly validity checks.

### Statistical analysis

Data was analyzed using SPSS 26.0 software. We first computed the crude rate of mental disorders in offspring, with 95% confidence intervals (CIs) using Wilson Score methods. To improve population generalizability, age- and sex-standardized rate were calculated through direct standardization against the demographic structure of China's 2020 national census,^19^ grouping participants into 5-year age intervals and by sex. Stratified analyses systematically evaluated variations across key sociodemographic and clinical strata. After performing multiple imputation, adjusted rate ratios were estimated using robust Poisson regression, adjusting for child sex, age, firstborn status, only-child status, household income, parental disease-birth timing relation, affected parental sex and age at childbirth. A sensitivity analysis was performed to assess result robustness by analyzing complete-case data and comparing with data from multiply imputed datasets. Multivariable analyses were performed for offspring under 17 years old. Initial univariable logistic regression identified associated variables at a liberal threshold (*p* < 0.10). Candidate variables were subsequently included in multivariable logistic regression models using backward stepwise selection (retention threshold: *p* < 0.05). All tests were two-tailed, with a significance level of *p* < 0.05.

### Ethics approval

Written informed consent de novo was obtained from all participants for the original national cross-sectional study. Ethics approval (No. 2021-1-13-7) was granted from the Institutional Review Board of Peking University Institute of Mental Health (PUIMH).

### Role of the funding source

The funding entities were not involved in any aspect of the study, including research design, data collection, analysis or interpretation, manuscript drafting or revision, or decisions regarding publication. The corresponding author maintained authority over data stewardship and assumes sole responsibility for publication decisions.

## Results

### Sample characteristics

The study encompassed 27,315 individuals living with schizophrenia who had offspring, including 9302 fathers (34.05%) and 18,013 mothers (65.95%). Fathers were slightly older than mothers (*p* < 0.001). Marital status differed significantly by sex, with mothers more frequently married or widowed and less commonly divorced (*p* < 0.001). Most patients resided in low-income households (<¥2000 monthly per capita), while mothers were disproportionately represented in extreme low-income households (<¥500 monthly per capita, 25.21% vs. 22.36%, *p* < 0.001). Multiparity rates (≥2 children) were significantly higher among mothers (30.48%) than fathers (22.87%, *p* < 0.001) (Table 1).

**Table 1:** Demographic characteristics of study participants (parents living with schizophrenia, n = 27,315).

	Male (n = 9302)	Female (n = 18,013)	X^2^/t	*p*-value
Age (Mean ± SD)	49.38 ± 7.26	48.06 ± 8.00	13.644	**<0.001**
Marital status: n (%)^a^	–	–	1248.767	**<0.001**
Single	27 (0.29%)	72 (0.40%)	–	–
Married	6507 (69.98%)	15,115 (83.91%)	–	–
Widowed	192 (2.06%)	805 (4.47%)	–	–
Divorced	2562 (27.55%)	1994 (11.07%)	–	–
Cohabitated	10 (0.11%)	25 (0.14%)	–	–
Household monthly income (RMB): n (%)^a^	–	–	34.379	**<0.001**
<500	1831 (22.36%)	3869 (25.21%)	–	–
500–1999	4199 (51.29%)	7851 (51.15%)	–	–
2000–5999	2020 (24.67%)	3380 (22.02%)	–	–
6000–10000	105 (1.28%)	194 (1.26%)	–	–
>10000	31 (0.38%)	53 (0.35%)	–	–
Number of children: n (%)	–	–	207.297	**<0.001**
1	7175 (77.17%)	12,522 (69.52%)	–	–
2	1984 (21.34%)	4878 (27.08%)	–	–
≥3	143 (1.54%)	613 (3.40%)	–	–

Bold type indicates statistical significance.

a Data not available for all patients.

### Parent-reported rate of mental disorders in offspring

Among 35,772 offspring, the crude rate of mental disorders was 2.68% (95% CI, 2.52%–2.85%). Schizophrenia spectrum disorders (SSD) were the most prevalent (1.43%, 95% CI, 1.31%–1.56%), followed by intellectual disability (ID, 0.59%, 95% CI: 0.52%–0.67%) and mood disorders (MD, 0.25%, 95% CI, 0.20%–0.31%) (Table 2). After age- and sex-standardization using China's 2020 census data, the overall rate (0–44 years) decreased marginally to 2.53% (95% CI, 2.01%–3.05%). Adult offspring (18–44 years) exhibited significantly higher rates (3.29%, 95% CI, 2.47%–4.11%) than children (0–17 years: 1.23%, 95% CI, 1.01%–1.44%) (Table 3).

**Table 2:** Parent-reported rates of mental disorders in study offspring (n = 35,772).

	Specific diagnosis	n	Reported rates (%)	95% CI (%)	
				Lower band	Upper band
Schizophrenia spectrum disorders	Schizophrenia	509	1.42	1.30	1.55
	Schizoaffective disorder	2	0.01	<0.01	0.03
Intellectual disability	–	210	0.59	0.52	0.67
Mood disorders	Depression	47	0.13	0.10	0.17
	Bipolar disorder	39	0.11	0.08	0.15
	Unspecified	4	0.01	<0.01	0.03
Others	Schizophrenia-like psychosis in epilepsy	16	0.04	0.02	0.07
	ASD	10	0.03	0.02	0.05
	ADHD	9	0.03	0.02	0.05
	Tic disorder	1	<0.01	<0.01	0.01
	Anxiety	6	0.02	0.01	0.04
	OCD	2	0.01	<0.01	0.03
	Personality disorder	1	<0.01	<0.01	0.01
Unclear diagnosis	–	103	0.29	0.24	0.35
Total	–	959	2.68	2.52	2.85

Note: ASD, Autism spectrum disorder; ADHD, Attention deficit and hyperactivity disorder; OCD, Obsessive-Compulsive Disorder.

**Table 3:** Age- and sex-standardized rates of study offspring.

	Age group	National population	Study population	Reported disorders (n)	Standardized rate (%)	95% CI (%)	
						Lower band	Upper band
Male	<18	158,416,523	5300	68	1.28	1.28	1.29
	18–24	54,906,394	3988	112	2.81	2.80	2.81
	25–29	48,162,270	4159	150	3.61	3.60	3.61
	30–34	63,871,808	4076	142	3.48	3.48	3.49
	35–39	50,932,037	1076	51	4.74	4.73	4.75
	40–44	47,632,694	41	1	2.44	2.39	2.49
Female	<18	139,239,421	4978	58	1.17	1.16	1.17
	18–24	48,447,415	3526	99	2.81	2.80	2.81
	25–29	43,685,062	3693	110	2.98	2.97	2.98
	30–34	60,273,382	3662	116	3.17	3.16	3.17
	35–39	48,080,895	915	27	2.95	2.95	2.96
	40–44	45,322,636	26	1	3.85	3.77	3.92

**Fig. 3:**
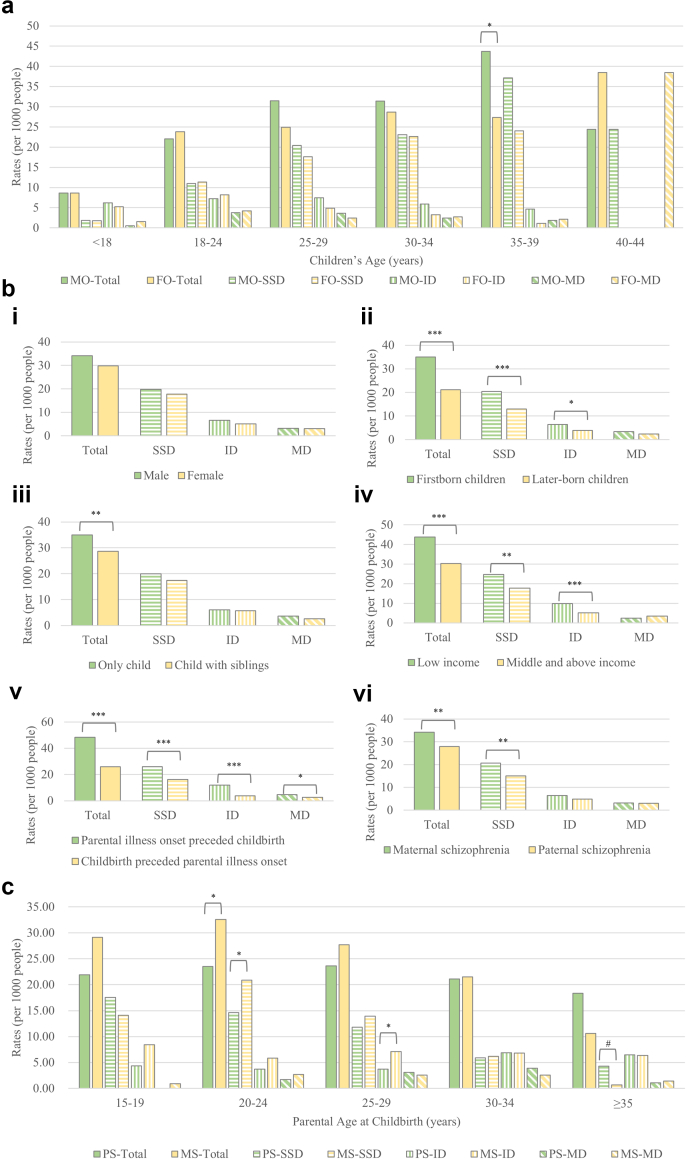
**Stratified analyses of parent-reported mental disorder rates among offspring of schizophrenia probands: Parent-reported rates by offspring sex and age (a), Parent-reported rates for subgroups (b), and Parent-reported rates by parental sex and parental age at childbirth age (c).** Note: 1) Visual symbols (consistent across all panels): Solid-colored bars = all mental disorders, horizontal-striped bars = schizophrenia spectrum disorders (SSD), vertical-striped bars = intellectual disability (ID), diagonal-striped bars = mood disorders (MD). 2) Statistical significance is denoted by asterisks: ∗*p* < 0.05, ∗∗*p* < 0.01, ∗∗∗*p* < 0.001, all based on chi-square tests. 3) Abbreviations: MO = male offspring; FO = female offspring; PS = paternal schizophrenia; MS = maternal schizophrenia. a. Highest rate was observed in 35–39 years. In this age group, total rates were significantly higher in MO than in FO (*p* < 0.05). b. i) Rates were numerically higher in MO than in FO, but no significant differences were observed. Significantly higher rates were observed in ii) first-born offspring for total (*p* < 0.001), SSD (*p* < 0.001), and ID (*p* < 0.05); and iii) only children for total and SSD (both *p* < 0.01); and iv) offspring from low-income households (<¥500/month per capita) for total (*p* < 0.001), SSD (*p* < 0.01), and ID (*p* < 0.001); and v) parental illness onset preceded childbirth for total, SSD, and ID (all *p* < 0.001). and vi) offspring from maternal schizophrenia group for total and SSD (both *p* < 0.01). c. Offspring of affected mothers aged 20–24 years at childbirth had significantly higher total and SSD rates than offspring of affected fathers at the same age ranges (*p* < 0.05). Offspring of affected fathers aged over 35 years at childbirth had a trend toward increased offspring risks for SSD (*p* = 0.064).

Stratified analyses revealed the highest rate of mental disorders in offspring aged 35–39 years, with males showing higher rates than females (4.74% vs. 2.95%, *p* = 0.040; Fig. 3a). Regression analyses identified familial risk gradients (Table 4). Results from both approaches were highly consistent, with substantial overlap in 95% CI and full consistency in statistical conclusions (Table S1). Parental disease characteristics had particularly prominent effects. Offspring risk increased by 86.0% when conception occurred post-parental illness onset (aRR = 1.86, 95% CI, 1.64–2.21, *p* < 0.001). Firstborn status (aRR = 1.67, 95% CI, 1.35–2.07, *p* < 0.001), low-income household (<¥500/month, aRR = 1.43, 95% CI, 1.24–1.66, *p* < 0.001), and being a male child (aRR = 1.14, 95% CI, 1.01–1.30, *p* = 0.038) significantly increased the risk. Sex-specific parental age effects were also observed. Offspring of affected mothers had a higher rate than those of affected fathers (aRR = 1.26, 95% CI, 1.10–1.45), especially when mothers were 20–24 years old at childbirth (X^2^ = 7.416, *p* < 0.01). Conversely, older paternal age (>35 years at childbirth) showed a trend toward increased offspring risks, particularly for SSD (X^2^ = 3.426, *p* = 0.064).

**Table 4:** Robust Poisson regression analyses of intergenerational transmission risk factors of mental disorders in study offspring.

	B	SE	*p*	aRR	95% CI	
					Lower band	Upper band
Child sex: Male	0.13	0.06	**0.04**	1.14	1.01	1.30
Firstborn	0.51	0.11	**<0.001**	1.67	1.35	2.07
Only-child	0.06	0.08	0.42	1.06	0.91	1.24
Lower household monthly income (<500 RMB per capita)	0.36	0.07	**<0.001**	1.43	1.24	1.66
Childbirth after parental schizophrenia onset	0.62	0.07	**<0.001**	1.86	1.64	2.11
Maternal schizophrenia	0.23	0.07	**0.001**	1.26	1.10	1.45
Child age, year	0.05	0.00	**<0.001**	1.06	1.05	1.06
Affected parental age at childbirth, year	0.03	0.01	**<0.01**	1.03	1.01	1.04

Bold type indicates statistical significance.

### Intergenerational transmission risk factors in children and adolescents

Among 9475 offspring under 17 years, the sex of the affected parent was marginally associated with offspring mental disorders (X^2^ = 2.886, *p* = 0.089). Therefore, subsequent analyses were stratified by parental sex. For offspring of mothers with schizophrenia, univariate analyses identified significant associations between offspring psychopathology and older age (*p* < 0.001), lower household income (*p* < 0.001), birth after maternal illness onset (*p* = 0.008), and prenatal antipsychotic exposure (*p* = 0.024) (Table 5). Multivariable analyses further revealed independent risk factors: birth after parental illness onset (OR = 2.48, 95% CI, 2.01–3.06, *p* < 0.001), lower household income (OR = 2.32, 95% CI, 1.93–2.80, *p* < 0.001), prenatal antipsychotic exposure (OR = 1.69, 95% CI, 1.38–2.08, *p* < 0.001), and older offspring age (OR = 1.11, 95% CI, 1.09–1.14, *p* < 0.001) (Table S2).

**Table 5:** Univariate analyses of intergenerational transmission risk factors of mental disorders in underage (<17 years) offspring.

	Maternal schizophrenia				Paternal schizophrenia			
	Offspring without mental disorders (n = 6329)	Offspring with mental disorders (n = 82)	X^2^/t	*p*-value	Offspring without mental disorders (n = 3037)	Offspring with mental disorders (n = 27)	X^2^/t	*p*-value
Age (Mean ± SD)	10.36 ± 4.48	11.92 ± 3.47	−4.036	**<0.001**	10.95 ± 4.31	12.65 ± 2.97	−2.938	**0.007**
Sex: Male n (%)	3223 (50.92%)	42 (51.22%)	0.003	0.96	1626 (53.54%)	18 (66.67%)	1.855	0.17
Education years (Mean ± SD)^a^	5.14 ± 3.44	4.76 ± 2.88	0.923	0.36	5.60 ± 3.45	5.76 ± 2.79	−0.22	0.83
Household monthly income <500 RMB per capita: n (%)^a^	1420 (27.64%)	35 (47.95%)	14.74	**<0.001**	604 (24.25%)	10 (40.00%)	3.329	0.07
Childbirth after maternal schizophrenia onset: n (%)^a^	3241 (51.21%)	54 (65.85%)	6.95	**0.008**	1288 (42.41%)	13 (48.15%)	0.361	0.55
Prenatal antipsychotic exposure: n (%)^a^	580 (17.78%)	16 (29.63%)	5.065	**0.024**	172 (13.21%)	2 (15.38%)	0.053	0.82
Primary caregivers^a^	–	–	5.724	0.13	–	–	9.909	**0.019**
Mother	784 (12.39%)	16 (19.51%)	–	–	773 (25.45%)	3 (11.11%)	–	–
Father	1574 (24.87%)	21 (25.61%)	–	–	362 (11.92%)	7 (25.93%)	–	–
Parents	2836 (44.81%)	28 (34.15%)	–	–	1292 (42.54%)	8 (29.63%)	–	–
Others	1135 (17.93%)	17 (20.73%)	–	–	610 (20.09%)	9 (33.33%)	–	–

Bold type indicates statistical significance.

a Data not available for all patients.

For offspring of affected fathers, univariate analyses identified significant associations between offspring psychopathology with older age (*p* = 0.007) and father-only caregiving (*p* = 0.019), and with a marginal trend for lower household income (Table 5). Multivariable analyses identified independent risk factors father-only caregiving (OR = 2.15, 95% CI, 1.50–3.09, *p* < 0.001), lower household monthly income (OR = 1.97, 95% CI, 1.40–2.67, *p* < 0.001), male sex (OR = 1.79, 95% CI, 1.29–2.50, *p* = 0.001), and older offspring age (OR = 1.11, 95% CI, 1.06–1.16, *p* < 0.001) (Table S3). Notably, dual-parent caregiving exhibited a protective effect for young offspring (OR = 0.75, 95% CI, 0.51–1.10, *p* = 0.105). Unaffected offspring showed higher rates of dual-parent involvement compared to affected peers (44.07% vs. 33.03%, *p* = 0.021).

## Discussion

This nationwide investigation of 35,772 offspring from 27,315 parents living with schizophrenia provides pivotal insights into intergenerational mental illness transmission patterns in China. Notably, the study addresses two longstanding gaps in global research, namely the scarcity of large-scale, population-based evidence from China, and the lack of robust quantification of sex-specific transmission dynamics. Our core findings directly confirm the familial risk elevation of schizophrenia in China, aligning with international evidence. Our validation of cross-cultural consistency in familial risk while highlighting region-specific features advances scientific understanding and provides an empirical basis for targeted interventions.

The standardized parent-reported rate of mental disorders (2.53%) in offspring aligned with international evidence of elevated familial risk,^16^ yet remained lower than estimates from Western cohorts.^20^^,^^21^ Several interconnected factors likely explain this discrepancy. First, our reliance on community sampling and parental self-report introduced inherent limitations, which was prone to missing offspring with mild symptoms or families not engaged in clinical services. This bias was amplified by inadequate support for families of individuals with schizophrenia in China.^22^ Most families have low mental health literacy and limited access to specialized care,^23^ and thus they may fail to recognize early-stage mental health issues. A separate study by our team utilizing active screening in a smaller subset of population (n = 1114) found significantly higher rates of depressive and anxiety symptoms in offspring than in their comparisons.^24^ Second, the child and adolescent subgroup had a far lower standardized rate (1.23%) than adult offspring (3.29%). Since many mental disorders have an onset in early adulthood, so younger offspring may not have developed symptoms yet. Additionally, youth-specific symptoms are often misattributed to age-related behavioral issues. Finally, sociocultural factors unique to China further contribute to underreporting. Beyond the well-documented stigma related to mental disorders in China,^25^ additional social contextual factors may play a role. Traditional family norms that prioritize ‘problem-solving’ over ‘medicalization’, lead parents to avoid labeling their children's struggles as ‘disorders’.^26^

Stratified analyses revealed critical variations in intergenerational transmission patterns of mental ill health. After controlling for confounding variables, male or firstborn children showed higher disease susceptibility, whereas only-child status no longer affected disease risk. This may reflect son preference and the one-child policy in China.^27^ Families were more likely to have an additional child (to have a boy) if their firstborn was female, which may explain the higher proportion of males among only-children. The elevated risk in offspring born after parental illness onset aligned with epigenetic hypotheses, which suggested active parental illness during childrearing might exacerbate genetic vulnerabilities through environmental pathways.^28^ In light of these findings, healthcare institutions should prioritize the provision of specialized resources (e.g., mental health counseling, financial assistance programs) and support networks for children and families in such contexts.

Distinct transmission patterns by parental sex were evident. Notably, females accounted for 47.18% of the 70,332 participants with schizophrenia enrolled in our original large-scale research regardless of reproductive status. In contrast, a significant sex disparity was observed in the subset of participants who had children, with females markedly outnumbering males (65.95% vs. 34.05%). Importantly, offspring of mothers with schizophrenia faced greater risk than those of fathers, but the underlying pathways differed significantly. Maternal schizophrenia conferred greater risk, which was associated with maternal-specific perinatal environmental exposures.^29^ By contrast, paternal age emerged as a risk factor in older patients, consistent with evidence linking advanced paternal age to de novo gene mutations and neuronal plasticity and morphology alteration.^30^ Notably, poverty stood out as the most impactful and persistent factor affecting parental and child mental health.^31^ We recommend that policymakers and clinicians prioritize structural inequalities, i.e. poverty-alleviated care access, in mental health interventions to end intergenerational risk transmission.

Our findings further revealed divergent pathways of maternal vs. paternal schizophrenia transmission during critical developmental periods, necessitating parental sex-tailored interventions. For maternal transmission, the pronounced association with peripartum pharmacotherapy and extreme poverty underscored a dual biological-environmental pathway.^32^ Biologically, maternal illness directly disrupted fetal neurodevelopment via antipsychotic-induced epigenetic alterations and immune-inflammatory cascades, exacerbated by household malnutrition.^33^ Socially, affected mothers experienced more forced separation, while stigma amplified their self-doubt and isolation, impairing their ability to recognize offspring psychopathology.^34^ Conversely, paternal transmission mainly resulted from postnatal caregiving deficits. Father-only households lacked maternal buffering, creating adverse parenting environments marked by emotional neglect. This aligns with research linking paternal schizophrenia to higher rejection rates.^35^ Globally, fathers with schizophrenia often withdraw from caregiving due to untreated symptoms. Sons are especially vulnerable due to their heightened sensitivity to paternal interaction quality.^36^ Previous studies suggest dual-parent caregiving conferred protective effects, and highlights the buffering role of stable family relationships in mitigating psychiatric risks.^37^ Targeted interventions such as the “Let's talk about children” program,^38^ which focuses on empowering parents with SMIs by enhancing caregiving skills, might address these gaps and reduce intergenerational transmission risk. Based on our findings and research evidence,^39^ we recommend that preventive interventions integrate social and familial level strategies more comprehensively to significantly improve intervention effectiveness.

The large-scale population-based cohort design combined with hybrid sampling methodologies offers novel insights into intergenerational transmission of mental health disorders in the Chinese population. However, findings should be interpreted in light of several limitations. Firstly, reliance on parent-reported offspring diagnoses introduced recall bias and underestimated rates induced by delayed recognition of offspring symptoms. Secondly, while our large sample size enhanced statistical power, it necessitated incomplete control for environmental confounders. We did not comprehensively evaluate all potential environmental confounders, including perinatal stress, childhood trauma, and substance use, which could also influence intergenerational risk. The lack of data on parents’ clinical severity limited the interpretation of study outcomes, since illness severity may directly influence risk and protective factors in their offspring—such as antipsychotic use during pregnancy and family caregiving structure. Thirdly, this study did not collect data on the ethnicity or race of participants. Since the 686 Program serves a diverse population across China, the inability to account for ethnic or racial variations may limit the generalizability of our findings. The results may also be less applicable to individuals with schizophrenia who are not registered in the system, unable to access services, or choose to discontinue care. Longitudinal studies are needed to fully elucidate the temporal relationships between risk factors and offspring morbidity. Future studies using standardized psychiatric assessments coupled with biological measures (e.g., polygenic risk score^40^) and transgenerational perspective^39^ could strengthen etiological insights into intergenerational transmission and early identification for psychological prevention.

In conclusion, this study offered valuable insights into the intergenerational transmission of mental disorders in China. The identified rates and risk factors highlight the complex interplay of genetic, environmental, and socioeconomic factors. Policy makers should pay special attention to improve environmental conditions for patients raising children. Early screening for mental health problems, especially in high-risk offspring, could enable timely intervention and prevention. There is also a need to address structural inequalities to break the cycle of intergenerational psychiatric morbidity. Promoting family-focused support programs and parenting skills training could help optimize caregiving environments and reduce the intergenerational risk of mental disorders.

## Contributors

The two co-first authors of this research (Zhi Sheng, Tianhang Zhou) have made equally important contributions to the research work. Zhi Sheng conducted literature review, developed the methodology, analyzed the data, and drafted the original manuscript. Tianhang Zhou contributed to the data validation, participated in the discussion of results, and revised it critically for important intellectual content. Xiaozhen Lv, Chunyu Du, Tingfang Wu, and Weiran Chen jointly designed the study and conducted experiments. Liping Wen, Xianmei Yang, Wencai Chen, Xuehong Ma, Hua Deng, Ling Ge, Changchun Zhang, Xu Hong, Rui He, Xiangdong Du, Lingyan Zhu, Hu Xiang, Sijing Chen, Jiachen Liu, Yongzhuo Ding, Guangming Liang, Yang Pan, Shujiao Ji, Zhengjiao Chang, and Nian Yuan jointly contributed to the data collection and validation. Lili Guan contributed to the study conceptualization and secured funding for the research. Hong Ma and Xin Yu supervised the project. All authors have read and agreed to the published version of the manuscript. Lili Guan and Xin Yu were the corresponding authors, had full access to all the data, and gave final approval of the version to be published.

## Data sharing statement

The data that support the findings of this study are available from the corresponding author upon reasonable request. The data are not publicly available due to ongoing research based on the dataset.

## Declaration of interests

We declare no competing interests.
