# Supplementary material for: Sex-differences in the intergenerational transmission of mental disorders among schizophrenia probands: familial risk and protective factors in a population-based study
Source: Lancet Reg Health West Pac. 2025 Nov 20;65:101750. doi: 10.1016/j.lanwpc.2025.101750 (PMC12681752; doi:10.1016/j.lanwpc.2025.101750)
Supplement: Supplementary Table S1 [file mmc1.docx]

**Supplemental Table 1. Comparison of Robust Poisson Regression Results between Multiple Imputation and Complete Case Analysis for Risk Factors of Mental Disorders in Offspring**

|  | **Multiple Imputation Analysis^*^** | | | **Complete Case Analysis** | | |
| --- | --- | --- | --- | --- | --- | --- |
|  | **aRR** | **(95% CI)** | ***p*** | **aRR** | **(95% CI)** | ***p*** |
| Child sex: Male | 1·14 | (1·01-1·30) | **0·04** | 1·15 | (1·00-1·31) | **<0·05** |
| Firstborn | 1·67 | (1·35-2·07) | **<0·001** | 1·69 | (1·33-2·14) | **<0·001** |
| Only-child | 1·06 | (0·91-1·24) | 0·42 | 1·02 | (0·85-1·20) | 0·85 |
| Lower household monthly income (<500 RMB per capita) | 1·43 | (1·24-1·66) | **<0·001** | 1·55 | (1·35-1·79) | **<0·001** |
| Childbirth after parental schizophrenia onset | 1·86 | (1·64-2·11) | **<0·001** | 1·88 | (1·64-2·15) | **<0·001** |
| Maternal schizophrenia | 1·26 | (1·10-1·45) | **0·001** | 1·24 | (1·07-1·45) | **<0·05** |
| Child age, year | 1·06 | (1·05-1·06) | **<0·001** | 1··06 | (1·05-1·07) | **<0·001** |
| Affected parental age at childbirth, year | 1·03 | (1·01-1·04) | **<0·01** | 1·03 | (1·01-1·05) | **<0·001** |
| Sample size | 35,772 | .. | .. | 29,663 | .. | .. |

^*^ Multiple imputation used an automated Fully Conditional Specification approach, including all analysis variables. With low missingness (child age: 0.9%; affected parent’s age at childbirth: 0.9%; household monthly income: 16.2%), 5 datasets were imputed. Results were pooled using Rubin’s rules after robust Poisson regression.
