# Supplementary material for: Sex-differences in the intergenerational transmission of mental disorders among schizophrenia probands: familial risk and protective factors in a population-based study
Source: Lancet Reg Health West Pac. 2025 Nov 20;65:101750. doi: 10.1016/j.lanwpc.2025.101750 (PMC12681752; doi:10.1016/j.lanwpc.2025.101750)
Supplement: Supplementary Table S2 [file mmc2.docx]

**Supplemental Table 2. Multivariable analyses of intergenerational transmission risk factors of mental disorders in underage (< 17 years) offspring of maternal schizophrenia**

|  | **B** | **SE** | ***p*** | **OR** | **95%CI** | | |
| --- | --- | --- | --- | --- | --- | --- | --- |
|  |  |  |  |  | | **Lower band** | **Upper band** |
| Age | 0·11 | 0·01 | **<0·001** | 1·11 | | 1·90 | 1·14 |
| Sex: Male | 0·01 | 0·10 | 0·90 | 1·01 | | 0·84 | 1·22 |
| Lower household monthly income (<500 RMB per capita) | -0·84 | 0·10 | **<0·001** | 2·32 | | 1·93 | 2·80 |
| Childbirth after parental schizophrenia onset | 0·91 | 0·11 | **<0·001** | 2·48 | | 2·00 | 3·06 |
| Prenatal antipsychotic exposure | 0·53 | 0·11 | **<0·001** | 1·69 | | 1·38 | 2·08 |
