# Supplementary material for: Sex-differences in the intergenerational transmission of mental disorders among schizophrenia probands: familial risk and protective factors in a population-based study
Source: Lancet Reg Health West Pac. 2025 Nov 20;65:101750. doi: 10.1016/j.lanwpc.2025.101750 (PMC12681752; doi:10.1016/j.lanwpc.2025.101750)
Supplement: Supplementary Table S3 [file mmc3.docx]

**Supplemental Table 3. Multivariable analyses of intergenerational transmission risk factors of mental disorders in underage (< 17 years) offspring of paternal schizophrenia**

|  | **B** | **SE** | ***p*** | **OR** | **95%CI** | | |
| --- | --- | --- | --- | --- | --- | --- | --- |
|  |  |  |  |  | | **Lower band** | **Upper band** |
| Age, year | 0·10 | 0.02 | **<0·001** | 1·11 | | 1·06 | 1·16 |
| Sex: Male | 0·58 | 0.17 | **0·001** | 1·80 | | 1·29 | 2·50 |
| Lower household monthly income (<500 RMB per capita) | 0·66 | 0.16 | **<0·001** | 1·94 | | 1·40 | 2·67 |
| Primary caregivers: Only Father | 0·77 | 0.18 | **<0·001** | 2·15 | | 1·50 | 3·09 |
